# Statistics of Hospitals and Out-Patient Service

**Published:** 1887-10

**Authors:** 


### STATISTICS OF HOSPITALS AND OUT-PATIENT SERVICE.

VETERINARY DEPARTMENT. UNIVERSITY OF PENNSYLVANIA,

September 1st, 1886, to September 1st. 1887.

| | Hospital. | Out Patient. |
|---|---|---|
| Abscess | 4 | 14 |
| Amputation. Legs dog | | 3 |
| " Tails dog | | 3 |
| " Tails horse | 10 | |
| Anæmia | 4 | 3 |
| Anarsarca | 3 | 4 |
| Arthritis | 1 | 1 |
| Atrophy. Muscular | | 2 |
| Bronchitis | 1 | 10 |
| " Chronic | 1 | 4 |
| Bruised feet | 5 | 6 |
| Bursa | 1 | 3 |
| Calculi. Vesical | 1 | 1 |
| " Of penis | 1 | 2 |
| Canker | 3 | 5 |
| Caries. Sup. Maxillary | 1 | 2 |

| | Hospital. | Out Patient. |
|---|---|---|
| Castration | 4 | 10 |
| Cataract | 3 | 8 |
| Catarrh. Auricular | 10 | 11 |
| Champignon | 1 | 3 |
| Chorea | 5 | 6 |
| Cicatrix. Painful | 1 | 1 |
| Collar boil | | 1 |
| Collection of sinuses | 6 | 8 |
| Congestion. Cerebral | 1 | |
| " of liver | | 2 |
| " of lung | 4 | |
| " of spine | 6 | |
| Conjunctivitis | 1 | 3 |
| Constipation | | 10 |
| Contracted heels | 6 | 35 |
| Contraction of flexor tendons | 4 | 2 |
| Contusions | 3 | 5 |
| Convulsions | 5 | 2 |
| Corns | 3 | 15 |
| " Suppurating | 1 | 2 |
| Crapaudine | 2 | 1 |
| Curb | 3 | 7 |
| Cyst. Anus | 1 | |
| " Mucous | 2 | |
| " Serous | | 2 |
| " Testicles | 1 | |
| Cystitis | 1 | |
| Degeneration. Fatty liver | 1 | |
| " Flexor tendons | 1 | |
| Diarrhœa | 2 | 3 |
| Dislocations. Femoral articulation | 1 | 2 |
| Dislocations. Eye | | 1 |
| Distemper. Dogs | 20 | 17 |
| Dropsy. Abdominal | | 2 |
| Dysentery | | 1 |
| Ears. Cut | | 1 |
| Eczema | 17 | 19 |
| Emphysema | 1 | 5 |
| Endocarditis. Mitral | 3 | 3 |
| Enterititis | 5 | 2 |
| Epilepsy | | 1 |
| Erythema | | 3 |
| Eyes. Removed | | 2 |
| " Artificial supplied | | 1 |
| Examination for soundness | 1 | 57 |
| Examination | 8 | 1 |
| Filaria. Hæmtooza | 1 | |
| Fistula. Parotid | 1 | |
| " 2d Phalanx | 1 | 1 |
| " Withers | 3 | 3 |
| Fleas | | 1 |
| Foreign Bodies. Bone in throat | | 1 |
| Foreign Bodies. In maxilla | | 1 |
| Foreign Bodies. In rectum | | 1 |
| Fractures. Femur | 2 | 3 |
| " Humerus | 1 | 3 |
| " Nasal bone | 2 | 2 |
| " Pelvis | | 2 |
| " Phalanx | 1 | |
| " Radius | 3 | 3 |
| " Ribs | 2 | |
| " Tibia | 9 | 8 |
| " Vertebræ | 1 | |
| Founder | 7 | 1 |
| " Chronic | 2 | |
| Gastritis | 2 | 7 |
| Gastro enteritis | 1 | |
| Glanders | | 12 |
| Goitre | 1 | 1 |
| Grease | 6 | 8 |
| Growth. Fungus | | 1 |
| Hæmaturia | | 1 |
| Helminths. Ascaridies | 1 | 15 |
| " Tænia | 2 | 4 |
| Hemorrhage | 1 | |
| Hoof deformed | 1 | 2 |
| Hydrophobia | 2 | |
| " Suspected | 1 | |
| Hysteria | 1 | 1 |
| Impaction of rectum | 2 | 2 |
| Inanition | 1 | |
| Indigestion. Chronic | 2 | 9 |
| " Intestinal | 2 | 16 |
| Injury. Maxilla | 1 | |
| Influenza | 4 | 11 |
| Ingrowing nails | | 2 |
| Interfering | | 4 |
| Iritis | | 2 |
| Keratitis | | 3 |
| Knuckling | | 1 |
| Laryngitis | | 4 |
| " Chronic | | 1 |
| Leucocythæmia | | 1 |
| Locomotor ataxia | 1 | |
| Luxation. Scapula humeral | 1 | |
| " Eye | 1 | 3 |
| Mange Sarcoptic | 17 | 30 |
| " Dematodect | | 2 |
| Marasmus | | 1 |
| Meningitis. Cerebro spinal | 4 | |
| Navicular disease | 5 | 4 |
| Necrosis | | 2 |
| Neuritis. Rheumatic | 1 | |
| Old age | | 1 |
| Opthalmia. Periodic | 1 | 8 |
| " Traumatic | | 3 |
| Osselet | | 3 |
| Osteo Porosus | 2 | 2 |
| Ostitis | | 1 |
| Paralysis | 2 | 3 |

| | Hospital. | Out Patient. |
|---|---|---|
| Paralysis Lumbar. Dog | 2 | |
| Paraphlegia | 1 | |
| Paronychia | | 1 |
| Pneumonia | 13 | 2 |
| " Chronic | 1 | |
| Periostitis | 5 | 7 |
| Peritonitis | 1 | 1 |
| Pharyngitis | | 1 |
| Pleurisy | 1 | |
| Poisoning | 1 | |
| Poll evil | 1 | |
| Pricked foot | 5 | 10 |
| Prolapsus. Rectum | 1 | |
| " Uterus | 3 | 1 |
| Prostrate enlarged | 1 | 1 |
| Quarter crack | 1 | 7 |
| Quittor | 9 | 24 |
| Rachitis | 2 | |
| Rheumatism | 8 | 4 |
| Ringbone | 11 | 15 |
| Roaring | 3 | 7 |
| Rupture. Muscular | 1 | 1 |
| Shoe boil | 2 | 3 |
| Shoeing | | 493 |
| Sidebone | 3 | 1 |
| Spavin | 13 | 38 |
| Spayed. Bitches | 10 | 4 |
| Splints | 6 | 8 |
| Sprain of knee | | 1 |
| Strain of fetlock | | 1 |
| " Suspensory | | 1 |
| " Ligaments | 1 | |
| " Muscles | 1 | |
| Sunstroke | | 2 |
| Suppurating sole | 1 | |
| Surfeit | | 1 |
| Stumbling | | 1 |
| Synovitis. Carpal sheath | 1 | 7 |
| " " Great sesamoid sheath | 2 | 5 |
| Synovitis. Tibio tarsal | 1 | 1 |
| " Rheumatic | 1 | |
| Taenia | 1 | 8 |
| Tetanus | 7 | |
| Teeth. Filed | | 37 |
| " Irregular operated | | 7 |
| " Removed | | 4 |
| Toe crack | 5 | 8 |
| Thrush | 7 | 10 |
| Tuberculosis | 2 | 7 |
| Tumors. Carcinoma | 1 | 2 |
| " Epithelioma | 5 | 6 |
| " Fibroma | 2 | 3 |
| " Lypoma | | 1 |
| " Lymphdenoma | | 1 |
| " Melanotic | | 3 |
| " " Sarcoma | | 1 |
| " Myxoma | | I |
| " Papiloma | | 1 |
| " Sarcoma | 4 | 2 |
| " Sebaceous | | 1 |
| Ulcers | 4 | 10 |
| " Chronic | 1 | |
| Urticaria | | 4 |
| Vertigo | | 1 |
| Wounds. Incised | 4 | 2 |
| " Lacerated | 7 | 13 |
| Total | 429 | 1298 |

